# TFDP3 as E2F Unique Partner, Has Crucial Roles in Cancer Cells and Testis

**DOI:** 10.3389/fonc.2021.742462

**Published:** 2021-10-20

**Authors:** Jiahao Huang, Yini Wang, Jinlong Liu, Ming Chu, Yuedan Wang

**Affiliations:** ^1^ Department of Immunology, School of Basic Medical Sciences, NHC Key Laboratory of Medical Immunology, Peking University, Beijing, China; ^2^ Department of Basic Medicine and Forensic Medicine, Baotou Medical College, Baotou, China

**Keywords:** TFDP3, HCA661, CT30, DP4, E2F-1, RB, pocket proteins, cancer-testis antigen

## Abstract

Transcription factor DP family member 3 (TFDP3) is a cancer-testis antigen, mainly expressed in normal testis and multiple cancers. *TFDP3* gene (Gene ID: 51270) is located on the chromosome X and shares a high degree of sequence homology with TFDP1 and TFDP2, which can form heterodimers with E2F family members and enhance DNA-binding activity of E2Fs. In contrast to TFDP1 and TFDP2, TFDP3 downregulates E2F-mediated transcriptional activation. During DNA damage response in cancer cells, TFDP3 is induced and can inhibit E2F1-mediated apoptosis. Moreover, TFDP3 is involved in cell autophagy and epithelial-mesenchymal transition. Regarding cancer therapy opportunity, the transduction of dendritic cells with recombinant adenovirus-encoding TFDP3 can activate autologous cytotoxic T lymphocytes to target hepatoma cells. Here, we review the characterization of TFDP3, with an emphasis on the biological function and molecular mechanism. A better understanding of TFDP3 will provide new insights into the pathological mechanisms and therapeutic strategies for cancers.

## Introduction

In 2002, the HCA661 was first discovered in our laboratory by screening human hepatocellular carcinoma-associated antigens with autoantibodies, which has been identified as a cancer-testis antigen. The gene-encoding HCA661 is located on X chromosome. The mRNA of HCA661 is predominantly expressed in testis and multiple tumor tissues, rarely or not expressed in normal tissues. Through DNA sequencing and sequence alignment, we found that HCA661 shares a high degree of sequence homology with a transcription factor DP (TFDP) family member—TFDP1, which suggests HCA661 is a member of the TFDP family. In order to maintain the consistency of TFDP family proteins, we named HCA661 using TFDP3 recommended by the Hugo Gene Nomenclature. In addition, HCA661 is also referred as DP4 or CT30 (1–3). E2F family plays a key role in cell proliferation and cell cycle regulation. In normal conditions, TFDP proteins work cooperatively with E2F proteins, so TFDP family exerts critical function in a wide variety of fundamental life processes by interacting with E2F family (4, 5). Similar to other TFDP family members, TFDP3 dimerizes with different types of E2F subunits to perform various functions. However, TFDP3 has significant differences with other TFDP family members. Unlike the TFDP1/E2F1 and TFDP2/E2F1 complexes that can recognize DNA sequences such as TTT(C/G)GCGC(C/G) which exist in the promoters of many key genes, TFDP3/E2F1 complex has little or no DNA-binding activity (3, 6). In addition, TFDP3 inhibits E2F1-mediated transcriptional activity, apoptosis, and cell cycle regulation. Interestingly, this is mainly achieved by a domain in the C-terminal region and four amino acids in the DNA-binding domain of TFDP3, which downregulates the activity of E2F1. Also, TFDP3 induces autophagy in a non-E2F-dependent manner (7). Moreover, TFDP3 covers important functions in an E2F-independent way. Since TFDP3 plays an important role in the onset and development of multiple cancer, it appears to be a promising therapeutic target for the discovery of new cancer treatments (8, 9). This review focuses on the characterization of TFDP3, the involvement of TFDP3 in basic cell life processes and cancer development, as well as the mechanism of TFDP3 in the regulation of E2F-induced activity. Finally, we highlight current and emerging clinical strategies in cancer progression and therapeutic resistance.

## The Characterization and Expression of TFDP3

**Figure 1:**
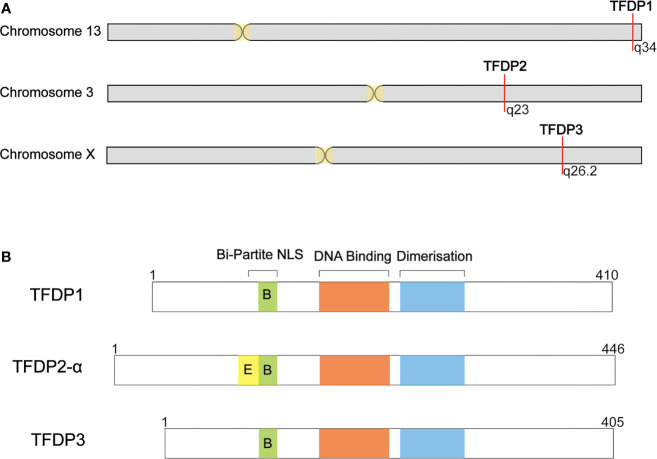
Summary of TFDP3 and other DP family members. **(A)** Location of DP family in chromosome. *TFDP1* and *TFDP2* locate in autosome, 13q34 and 3q23, respectively. Differently, TFDP3 locates in X chromosome, q26.2. **(B)** Structural comparison of DP family members. DP proteins share a conserved DNA-binding and dimerization domain. TFDP2-α has integrated Bi-Partite NLS; TFDP1 and TFDP3 have defective Bi-Partite NLS. The length of proteins is indicated.

**Figure 2:**
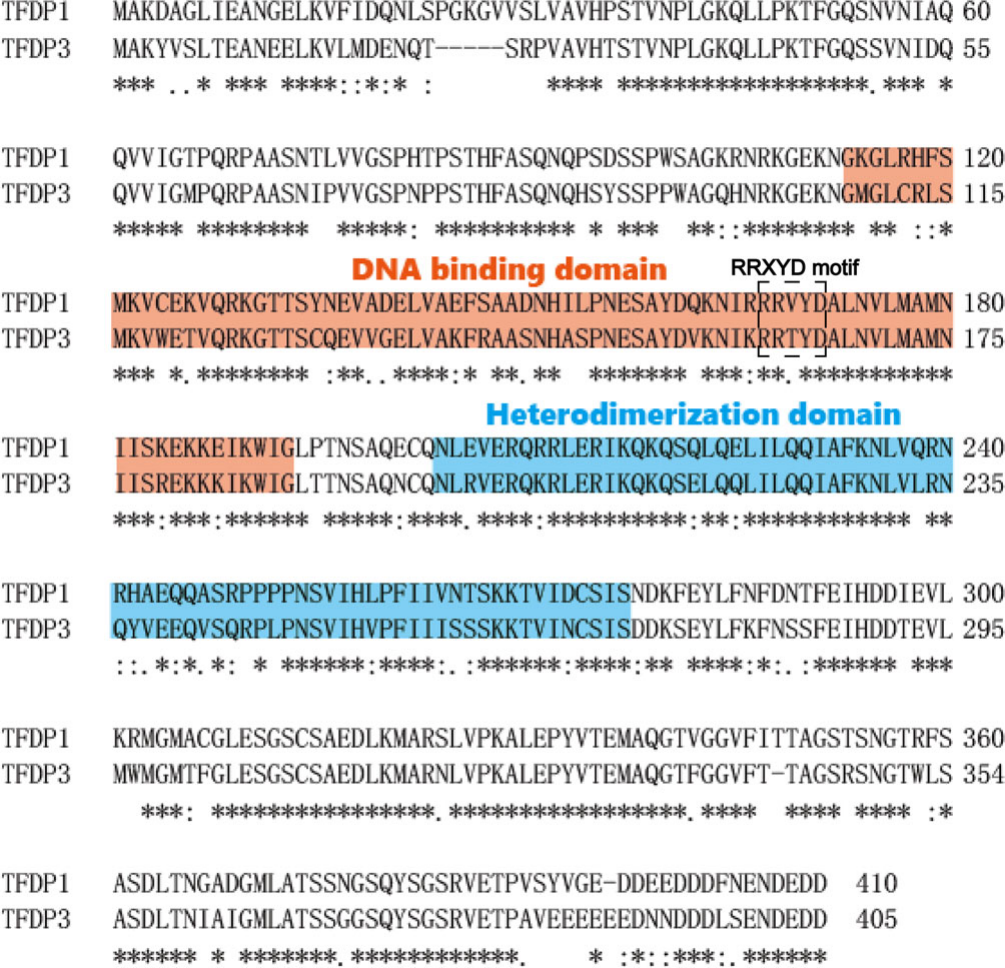
Amino acid sequence alignment of TFDP3 and TFDP1. Complete sequence of TFDP3 and comparison with TFDP1 using single-letter amino-acid code. Identical residues are indicated by “^*^” and similar residues by “•,” and the missing residues with a dash. The spanning of the DNA-binding domain is labeled in red, and the heterodimerization domain is labeled in blue. The RRXYD DNA recognition motif is circled by dotted lines.

When blasting the sequence of TFDP3, we found that it shares a homology of 88% in nucleotides and 73% in amino acids with TFDP1 (**Figure 1**). Furthermore, the *TFDP3* gene is located on chromosome X, q26.2 (1). Moreover, the overall length of *TFDP3* gene is 8,680 bp, it has only one exon and the mRNA length is 1,680 bp, encoding a 405-amino acid protein. In addition, the whole structure of TFDP3 is similar to that of TFDP1. Similarly, TFDP3 has the conserved DNA-binding domain and heterodimerization domain, which are typical in the TFDP family and are necessary for exerting functions (**Figure 2**). It is worth noting that an RRXYD E2F DNA recognition motif is also conserved in DNA-binding domain; however, the conformation in TFDP3 is RRTYD which is different to RRVYD in TFDP1 (2, 3). Moreover, they have a similar C terminus. Interestingly, TFDP3 mainly exists in *Homo sapiens* and other primates. However, TFDP1,2 can be identified in human, mouse, rat, dog, and other species. In a way, this may indicate that TFDP3 might generate at a later time in evolution (3). Although TFDP3 is not identified in mice, it is interesting to overexpress TFDP3 in mice normal cells. Maybe it can help to reveal more functions of TFDP3 and verify the published results.

The mRNA expression of TFDP3 was determined by RT-PCR; we found its mRNA was substantially expressed in normal testis and weakly expressed in normal pancreas. The TFDP3 mRNA is highly expressed in certain types of cancers such as hepatocellular carcinoma. The characteristic of TFDP3 is consistent with cancer-testis antigens, which has features including: (1) predominant mRNA expression in testis and certain types of cancers and (2) genes encoding cancer-testis antigens located on X chromosome (1). At the protein level, we also identified that TFDP3 appears in the malignancies of certain organs, such as the prostate, pancreas, and breast, through the immunohistochemistry in tissue microarray (10). This expression profile of TFDP3 is quite different from that of homologous TFDP1 which is ubiquitously expressed in a wide variety of human and murine tissues, while TFDP2 has a restricted expression pattern and several splice variants (11, 12). In terms of subcellular location, we detected that when TFDP3 is expressed without E2Fs, it is located mainly in the cytoplasm. It is novel that when TFDP3 is coexpressed with E2F1-3, it translates to the nucleus (3). It is well established that E2F1-3 are transcriptional activators. It is likely that TFDP3 represses the E2Fs transcriptional activity through intracellular translocation (13, 14). Another group of researchers stated that TFDP3 is mainly present in the nuclei of transfected cells, at the contrary of the cytoplasmic distribution of TFDP1 (2). We hypothesized that the reason for the aforementioned phenomenon is that TFDP3 is expressed variably in different cell cycle phases. Therefore, we implemented thymidine double repression method to induce cell synchronization to G1/S/G2. As expected, we verified that TFDP3 is expressed in the nucleus mainly at the end of mitosis phase, diffusively in G1 phase, and in the cytoplasm in G2 phase. Moreover, we detected that TFDP3 is highly expressed in S phase by Western blot of synchronous cells (15). Interestingly, a study of TFDP3 in prostate cancer emphasized that TFDP3 was coexpressed with E2F1. Furthermore, TFDP3 expression increased when coexpressed with E2F1. They reasoned that a binding site of E2F is present in the upstream of promoter region of TFDP3 (16). Furthermore, a research focused on the effect of DNA damage showed that TFDP3 can be induced by DNA damage in different cell types and may play a crucial role in mediating the cell cycle effects of DNA damage (17).

Since TFDP3 is expressed highly in testis, it is worthy to study the functions of TFDP3 in testis. Moreover, the overexpression of TFDP3 in normal cells should be conducted, in order to discover the roles of TFDP3 in tumor development. As already reported, many cancer-testis antigens are normally silenced by DNA methylation in most somatic cells, and DNA methylation inhibitors can reactivate the silenced genes (18). Whether the expression of TFDP3 is modulated by DNA methylation remains to be revealed.

## Physiology of TFDP3

Despite that TFDP3 has been identified as a member of the TFDP family, it exhibits significant differences with TFDP1 and TFDP2. The TFDP family is widely known to form heterodimers with E2Fs in order to exert their DNA-binding function coordinately. Accordingly, TFDP3 retained the capacity to bind E2F proteins. Although TFDP3 has been confirmed to interact with E2F1-6 *in vitro* and *in vivo*, it is necessary to further explore the pathophysiological effect made by the binding of TFDP3 with the other E2Fs. Similar to the TFDP1/E2F1 complex, TFDP3/E2F1 heterodimer could form ternary complexes with pRb (2). Intriguingly, the DNA-binding activity of the TFDP3/E2F1 heterodimer on the E2F DNA recognition site was considerably lower than the TFDP1/E2F1 heterodimer. Moreover, we found that exogenous TFDP3 expression resulted in no significant alteration in binding activity, suggesting that E2F-TFDP3 complexes were defective in DNA binding (2, 3). It was demonstrated that TFDP3 can bind to E2F-regulated genes in the presence of E2F1 by the fact that TFDP3 localized on E2F sites in the E2F1 and Cdc2 promoters, as detected by ChIP analysis, and the two genes were regulated by E2F pathway (19). Therefore, TFDP3 localized to endogenous E2F target genes. Furthermore, TFDP3 negatively regulates E2F target genes through modulating the access of E2F1 to chromatin. However, the association between TFDP3 and related promoters is weaker than the level of TFDP1 (2). It is still unknown whether this results in defective DNA binding or not.

The transcription activity of the different combinations of E2F and DP was examined through luciferase reporter assay; in sharp contrast to the synergistic effect of TFDP1 and TFDP2, TFDP3 exerts an inhibitory effect on E2F-mediated transcriptional activity (3, 20). Another group showed similar results, demonstrating that coexpression of TFDP1 enhances the E2F transcriptional activity, whereas the effect of TFDP3 is to reduce the level of E2F transcription. Importantly, four critical amino acids within the DNA-binding domain confer the suppressed effect of TFDP3. However, a research group stated that DNA binding is not required for the transcription effects of TFDP3, while the C-terminal region, outside the DNA-binding domain, is indispensable for the negative control of E2F1 activity (2, 3). Since TFDP3 operates negative regulation of E2F1 in a pRb-independent manner, it implies that there is an alternative biochemically distinct pathway for downregulating E2F1 activity. It remains unknown whether the DNA-binding defect of E2F1-TFDP3 complex is related to their inhibited transcriptional activity. One hypothesis of the mechanism by which TFDP3 repressed E2F-dependent transcription is that TFDP3 functions as a competitive inhibitor of TFDP1.

E2F family appears to play a pivotal role in coordinating events connected with proliferation and cell cycle arrest, dictated by the E2F subunit composition and interaction with pocket proteins (19, 21). As a member of E2F subunit, several researches have shown that TFDP3 is involved in the regulation of cell cycle. Overexpression of E2F3 leads to increase the proportion of cells in the S phase, and more cells entered into the S phase when cotransfected with TFDP1. However, this effect is decreased when cotransfected with TFDP3. Correspondingly, a study showed that the depletion of TFDP3 in multiple cell lines caused a decrease in the proportion of G1 cells, with increased proportion of S and G2/M cells (17). Therefore, TFDP3 functions as an inhibitor of E2F-regulated cell cycle progress. Accumulated evidences showed that knocking down the TFDP3 in L-02 and HepG2 cell lines by siRNA interference, the cells are blocked in the S phase, consequently TFDP3 participates in the process of G1 phase entry into S phase (15). Furthermore, the DNA-binding activity of TFDP3 is required for the cell cycle effects (2). Since the competition with TFDP1 results in the downregulation of cell cycle progress, it is reasonable to hypothesize that TFDP3 directs the E2F-targeted genes involved in G1 progression. Thus, it is necessary to identify the targeted genes of TFDP3 in the future.

In the condition of chemotherapy, ultraviolet irradiation, or serum deprivation, which leads to DNA damage, E2F1 has the property to induce cell apoptosis which could abolish the aberrant cells timely and maintain the stabilization of chromosomes (22, 23). Intriguingly, TFDP3 is also DNA damage responsive, and DNA damage favors formation of the TFDP3/E2F1 complex. In contrast to TFDP1 and E2F1, TFDP3 reduces the cell apoptosis (17). Studies showed that E2F family of transcription factors is extremely involved in apoptosis, through the regulation of many apoptosis-related genes (19). Several evidences indicate that the transcription factors TFDP1 and TFDP2 as binding partners for E2F, although they may not have a biological function on their own, are indispensable for regulating E2F activity and play a central role in cellular functions such as apoptosis. TFDP3 has similar characteristic of TFDP1 and TFDP2, as the capacity to regulate cell apoptosis. However, TFDP3 exhibits a repressive modulation of E2F-induced apoptosis, in contrast to the other two TFDP members (24). This further confirms TFDP3 as a negative regulator of E2F functions. Further evidences showed that TFDP3 could be induced by E2F1 and expressed in coordination with E2F1, it can play a crucial role in prostate cancer cell survival by inhibiting apoptosis induced by E2F1 (16). Recently, we clarified that the downregulation of TFDP3 by RNA interference reversed chemoresistance through restoring E2F1 activity, which leads to increased levels of p53, p73, and associated proapoptotic target genes (25). The pathways of E2F-induced apoptosis includes the p53-dependent and p53-independent pathways (26). Then, it is feasible that TFDP3 mediates the E2F apoptosis by the two pathways. Indeed, studies showed TFDP3 reduced the p53 accumulation caused by E2F1 transfection and changes the expression level of p53-targeted genes (27). Weather TFDP3 interferes the E2F-mediated p53-independent way should be further explored.

Several studies have focused on the effect on E2F transcriptional activity and E2F-mediated cell cycle regulation by TFDP3. However, the important physiological functions of TFDP3 are not limited to these. Recently, we reported TFDP3 expression can trigger cell autophagy by upregulating the expression of autophagic key protein LC3 as well as the number of autophagosomes in the condition of etoposide treatment (28). Interestingly, this facilitated effect of TFDP3 is opposite to other suppression effects discussed above. It is well known that TFDP3 exerts the regulating function through inhibiting the E2F1 activities in the form of TFDP3/E2F1 heterodimer. However, we clarified that p53 acts as a critical bridge connecting TFDP3 expression and inducing autophagy in breast cancer cell line. The level of TFDP3 expression is inversely proportional to p53 expression, while in line with protein LC3. Hence, TFDP3 could promote cell autophagy by affecting the expression of p53 directly or indirectly (27, 28). However, the clear mechanism of this regulation remains open to study.

## The Molecular Mechanisms of TFDP3

E2F proteins are transcription factors believed to be involved in the activation of genes required for cellular proliferation (29, 30). The E2F/TFDP complexes coordinate events in the cell cycle by its cyclical interactions with important cellular proliferation regulators, such as the retinoblastoma tumor-suppressor gene product (Rb) and related proteins (31). Transcriptional control during the G1/S transition is important for the regulation of cell cycle progression and the TFDP1/E2F1 complex can increase entry into S phase (19, 32). TFDP3 as a TFDP family member is demonstrated to have a role in the process of cell cycle with E2Fs. Unlike TFDP1- and TFDP2-enhanced E2F binding with downstream target genes, E2F/TFDP3 complexes fail to bind with DNA (3). Furthermore, the failed DNA-binding activity of TFDP3/E2F1 complex is required for cell-cycle effects (2). Consequently, TFDP3 may act as a repressed factor of cell cycle progress. Indeed, knocking down the endogenic TFDP3 increases the proportion of cells in S phase. The properties of the TFDP3/E2F1 complex appear to be more related to the induction of G2 cells (2, 15).

**Figure 3:**
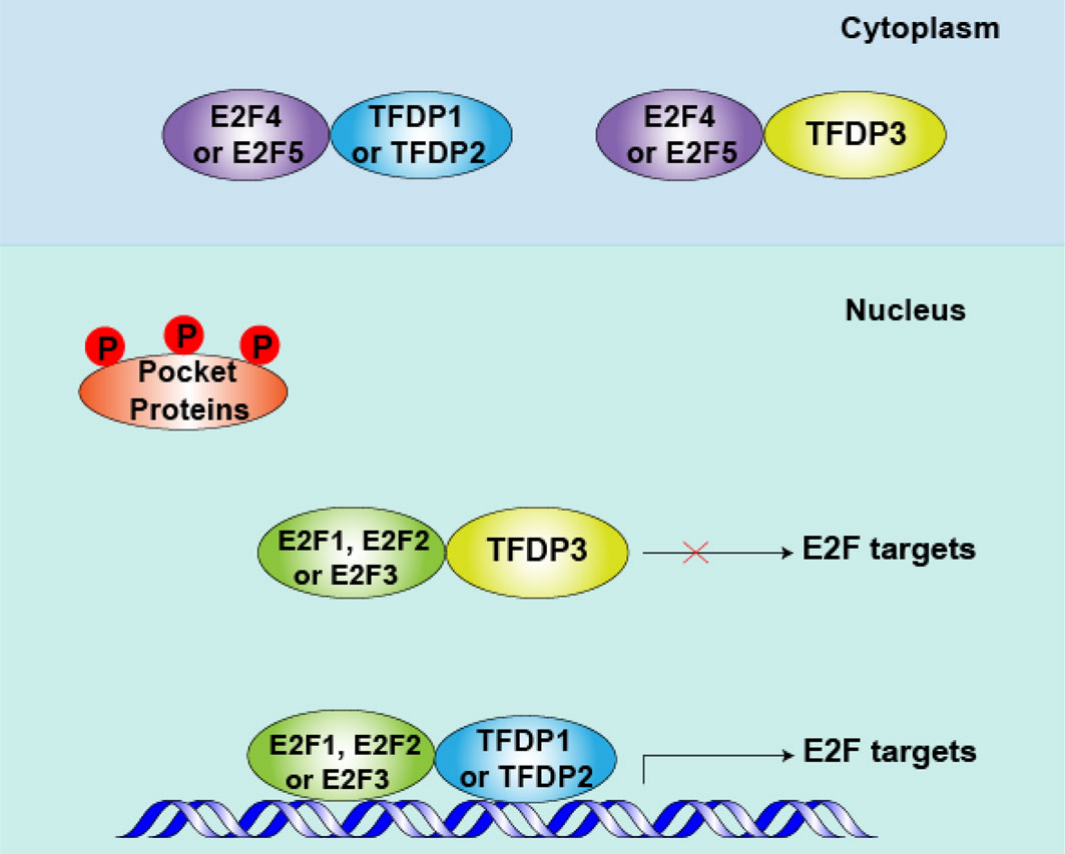
Role of TFDP3 in mid-to-late G1 phase. After pocket proteins (RB, p107 and p130) are phosphorylated by cyclins (CCNs) and cyclin-dependent kinases (CDKs) in early G1 phase, the E2F-TFDP complexes separate from pocket proteins in mid- to late-G1 phase. Meanwhile, repressive E2F4 and E2F5 are shuttled to the cytoplasm, and the activator protein E2F1-3 levels peak at this phase. When TFDP3 is present, the DNA-binding and transcriptional activities of E2F1-3 are inhibited. Consequently, the cell cycle-related protein is down produced and cell cycle can be arrested in G1 to some extent.

In addition, the spatial location of transcription is essential for their function. Most E2Fs, TFDP1 and TFDP2 are predominately nuclear, but E2F4 and E2F5 show cell cycle-specific localization (33–35). Furthermore, we found that subcellular location of TFDP3 is cell cycle specific, it is expressed in the nucleus in G1 phase and at the end of mitosis, in the cytoplasm in S phase and G2 phase (15). Thus, in quiescent cells, repressed E2F4 and E2F5 interacted mainly with TFDP1 or TFDP2, binding with E2F-targeted promoters and blocking the transcription of cell cycle-related genes. In cycling cells, after the phosphorylation and inactivation of pocket proteins in mid-to-late G1 phase, E2F4 and E2F5 are shuttled to the cytoplasm (36, 37). At the same time, E2F1-3 superiorly combine with the target promoters, when partner is TFDP1 or TFDP2, the cell cycle-related genes being activated, promoting the G1-S transition (6, 12). However, when TFDP3 is present, the E2F1-3/TFDP3 complexes fail to bind with E2F targets and the E2F activity level decreases. If the threshold of E2F activity cannot reach the restriction point of G1-S phase transition, the cell cycle is arrested in G1 phase (38, 39) (**Figure 3**).

**Figure 4:**
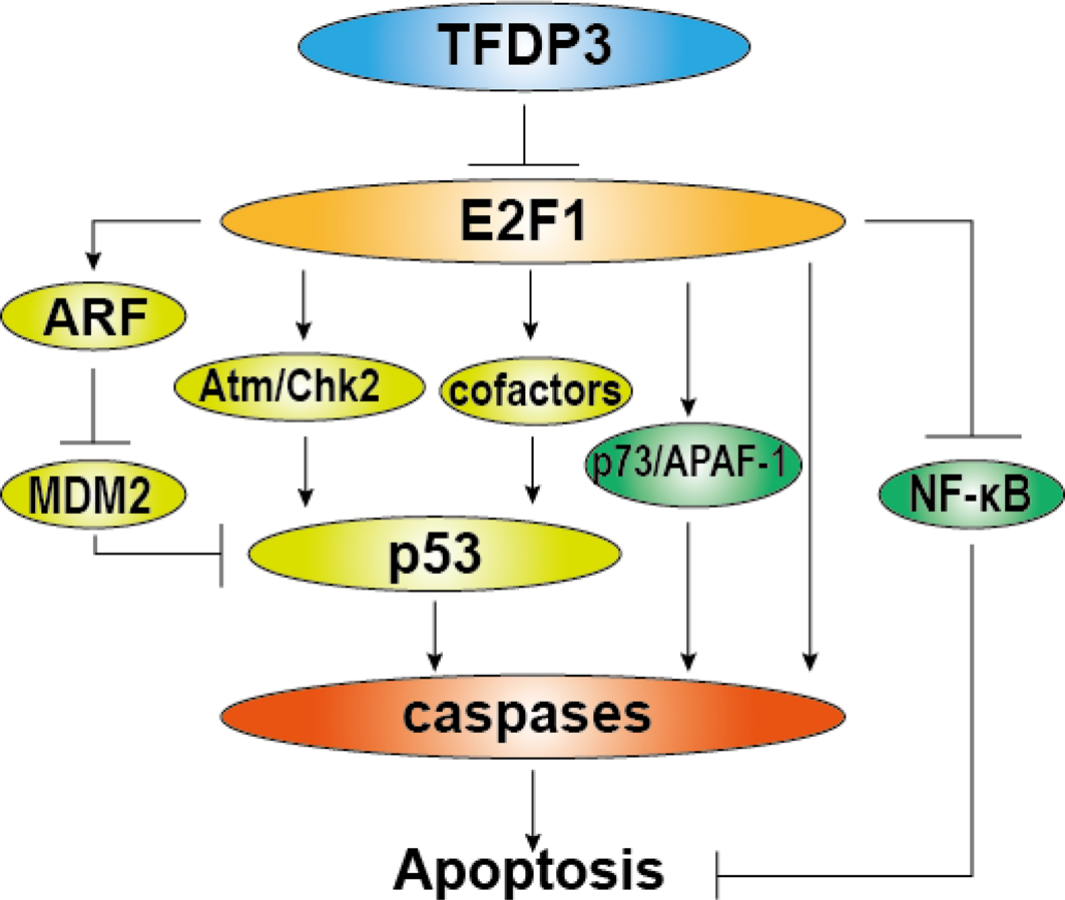
Role of TFDP3 in E2F1-induced apoptosis. The diagram summarizes general activation pathways of E2F1-induced apoptosis and the repressive role of TFDP3. The light green part represents p53-dependent mechanisms, and the green part represents p53-independent mechanisms. In the present TFDP3, the effect of E2F1-induced apoptosis is subverted.

Besides, we and others have found that TFDP3 inhibits the apoptosis induced by E2F1 (16, 27). In addition to the important roles in cell cycle regulation, E2F1 has a significant function in the induction of cell apoptosis (40–42). Moreover, high-level E2F1 tends to make cell apoptosis (43). In general, the E2F1-induced apoptosis is classified into p53 dependent and p53 independent. The p53-dependent pathway includes transactivation of ARF by E2F1. ARF binds to Mdm2 which can ubiquitinate and degrade p53, thus the level of p53 is increased. Moreover, E2F1 can enhance the p53 activity by Atm/Nbs1/Chk2 pathway. Inducing the proapoptotic cofactors of p53, such as ASpp2, JMY, and TP53INP1 can also enhance apoptosis (26, 42, 44, 45). In the situation of p53-independent pathway, E2F1 induces the transcription of the p53 homologs p73, Apaf-1, and caspase. Meanwhile, E2F1 inhibits activation of antiapoptotic NF-κB by downregulating TRAF2 protein level (46–48). As repressed partner of E2F1, TFDP3 can block the E2F1-mediated apoptosis (**Figure 4**). Furthermore, we found that TFDP3 elevates p53 expression and the mRNA expression of p53 target genes such as Bax, Puma, Noxa, and Bid, indicating that p53 pathway has an important role in TFDP3-repressing apoptosis (27, 28). It should be further investigated whether TFDP3 directly affects the targets p53 and p53.

## Role of TFDP3 in Disease

TFDP3 is involved in cell growth, apoptosis, and regulation of E2F activity. Thus, as a pivotal collaborative protein of E2F, TFDP3 exerts a crucial role in diseases, particularly in cancers as detected in immortalized human hepatocyte line L-02 and hepatocellular carcinoma cell line HepG2 at mRNA and protein level. Moreover, silencing the TFDP3 tends to increase the rate of apoptosis of cells. These results indicate that TFDP3 is involved in the process of hepatocellular carcinoma formation in a way of regulating the cell cycle and apoptosis (15). Also, TFDP3 participates in hepatocellular carcinoma through interfering with HIF-2α and modulating the cell apoptosis mediated by the E2F1 pathway. It was demonstrated that HIF-2α significantly increases apoptosis by repressing expression of TFDP3 and induces apoptosis through the TFDP3/E2F1 pathway (49). Furthermore, TFDP3 was increased in the minimal residual disease (MRD)-positive childhood T-ALL patients, and the silencing of TFDP3 expression can benefit in overcoming chemoresistance after chemical treatment (25).

TFDP3 is expressed in breast cancer cell lines, but it is preferentially expressed in mesenchymal then in luminal types of breast cancer. In this kind of tumors, since TFDP3 functions as a regulator of the epithelial-mesenchymal transition (EMT), it significantly increases their migratory and invasive activity (10). As we all know, autophagy can help cells cope with metabolic stress and remove erroneously folded proteins, defective organelles, or intracellular pathogens to prevent the accumulated aberrance of cells. Unfortunately, TFDP3 expression can trigger autophagy by upregulating the expression of autophagic key proteins and increasing the number of autophagosomes during chemotherapy of malignant tumors, therefore TFDP3 attributes chemical resistance by repairing damaged DNA of cancer cells (28). In addition, a study conducted by Xiaoke Hao suggested that TFDP3 is involved in prostate cancer cell survival by suppressing cancer cell apoptosis induced by E2F1 (16).

## TFDP3-Directed Therapeutics

The TFDP3 protein is immunogenic and capable of inducing an antibody response. We constructed a recombinant adenovirus-expressing TFDP3 and then transduced immature DCs; excitingly, the approach can help to induce DC maturation and these DCs can activate T cells to target hepatoma. Therefore, the recombinant adenovirus may have a potential usage in liver cancer immunotherapy (50). On this basis, an assay has been done to search for immunogenic peptides of TFDP3, and two HLA-A*0201-restricted peptides, H110 and H246, have been identified by employing bioinformatics analysis and CD8^+^ T cell IFN-γ ELISPOT. The two peptides of TFDP3 are hence attractive candidates for human cancer immunotherapy (8). Moreover, it was demonstrated that mRNA vaccine could elicit mutation-specific T-cell responses against predicted neoantigens (51).

As we all know, transcriptional master regulators exert a critical role in cancer genetic networks, and TFDP3 and other transcription factors have been identified in gene regulatory network of primary breast cancer. In addition, sets of genes controlled by TFDP3 are involved in processes that are well-known hallmarks of cancer, which inferred that using TFDP3 as a biomarker for cancer diagnosis could be feasible (52). Intriguingly, TFDP3 confers chemoresistance in minimal residual disease within childhood T-cell acute lymphoblastic leukemia, and downregulation of TFDP3 by RNA interference sensitizes cancer cells to combinational chemotherapy. Thus, it is possible that TFDP3 is a potential gene therapy target for residual cancer (25). As molecular chaperone of E2F, TFDP3 exerts transcriptional control during the G1/S transition in regulating cell cycle progression coordinately with E2F proteins. It has been approved that TFDP3 can promote cancer occurrence by modulating the cell cycle. In a previous work, it was found that the cell cycle would be arrested in S phase by knocking down the TFDP3. In this case, TFDP3 could be a potential target for tumor therapies. Interestingly, it has been reported that TFDP3 is expressed in breast cancer and acts as a transcription factor during epithelial-mesenchymal transition. It is well known that EMT is considered the initial step of distant organ metastasis because it induces epithelial cells to lose their polarity and intracellular adhesion forces but gain the ability to migrate through the basement membrane (10, 15). Accordingly, silencing TFDP3 may hinder the exacerbation of breast cancer by blocking the EMT. These results provide an opportunity to consider TFDP3 as a candidate promising target for breast cancer therapies.

Moreover, overexpression of TFDP3 can trigger cell autophagy during chemotherapy of tumors. The damage of DNA and organelles caused by chemotherapy can be repaired by autophagy. Thus, the siRNAs targeting TFDP3, which makes it possible to reduce tumor drug resistance, can be used to prolong the patient’s survival. Furthermore, a research suggested that TFDP3 is involved in prostate cancer cell survival by suppressing apoptosis (16, 28). Therefore, reducing the level of TFDP3 is an ideal way to restore the E2F physiological function and thereby recover the normal apoptosis rate in cells, which could improve the survival of patients with prostate cancer and other carcinomas.

As already reported, TFDP3 belongs to cancer-testis antigens which are mainly expressed in germinal and cancer cells; they can be found in some diseases as a prerequisite for downstream immune target-discovery projects. Recently, a study showed that the expression of TFDP3 varies significantly after a cycle of azacitidine treatment in myelodysplastic syndromes and chronic myelomonocytic leukemia through an *ad hoc*-targeted RNA sequencing. Consequently, TFDP3 emerges as a main candidate for immunotherapeutic targeting (9). By searching for EMT-related genes in gastric adenocarcinoma, a group analyzed a contribution of risk score formula using GO and KEGG enrichment analyses from the TCGA and GEO databases. They identified six pivotal EMT-related genes including TFDP3, which provide a potential prognostic signature for predicting prognosis of gastric adenocarcinoma patients (53).

However, it still remains unclear whether TFDP3 affects the immune system or not; therefore, side effects should be considered when targeting TFDP3. To date, no human cancer clinical trials have been conducted to target TFDP3. Although there is no homologous *TFDP3* gene in mice, other primates such as rhesus and chimpanzee have a human *TFDP3* homolog. Therefore, rhesus or chimpanzee can be a suitable animal model candidate for trials in the future. In addition, despite that TFDP3 was confirmed to be a potential therapeutic target in cancer, there are still no drugs targeting TFDP3 for cancer therapy. It is extremely necessary to find or synthesize chemical molecules against TFDP3 in the future.

## Conclusions and Perspective

In conclusion, TFDP3 is a member of the TFDP family and belongs to cancer-testis antigen, which exerts vital physiology function. Despite TFDP3 exhibits certain similarities with other members of the TFDP family, it also possesses a multitude of differences. These functional conversions result from four key amino acid substitutions in the DNA-binding domain and a C-terminal region of TFDP3. Importantly, TFDP3 is regarded as the inhibitor of E2F1 molecules. Since E2Fs are critical transcription factors involved in vital cell fundamental activities such as cell cycling, DNA replication, cell proliferation and differentiation, or other important physiological activities, TFDP3 exerts a pivotal role in mediating a great deal of physiology activities through forming a heterodimer with E2F subunits, and thereby reducing the DNA-binding activity of TFDP/E2F complexes. TFDP3 suppresses E2F1-mediated transcriptional activation and cell cycle progression and inhibits E2F1-induced, p53-mediated apoptosis. However, TFDP3 can induce cell autophagy, which is different with other repressive functions. In the aspect of pathology, TFDP3 is a cancer-testis antigen and is expressed abnormally in a host of malignant tumors. It is also clear that TFDP3 confers chemoresistance in minimal residual disease and regulates epithelial-mesenchymal transition in breast. Importantly, TFDP3 is an immunogenic protein and can help to activate T cells. Finally, *TFDP3* is a cancer-related gene showing a pivotal role in certain cancers and potential prognostic and therapeutic signature for carcinomas. The ongoing intensive investigation into TFDP3 will be crucial for the discovery of new therapeutic approaches to counter the disease.

## Author Contributions

JH wrote the manuscript and drew the pictures. MC and YDW contributed to conception and design of the review. All authors contributed to manuscript revision and read and approved the submitted version.

## Funding

This study was supported by the National Natural Science Foundation of China (81603119) and Natural Science Foundation of Beijing Municipality (7174316).

## Conflict of Interest

The authors declare that the research was conducted in the absence of any commercial or financial relationships that could be construed as a potential conflict of interest.

## Publisher’s Note

All claims expressed in this article are solely those of the authors and do not necessarily represent those of their affiliated organizations, or those of the publisher, the editors and the reviewers. Any product that may be evaluated in this article, or claim that may be made by its manufacturer, is not guaranteed or endorsed by the publisher.

## References

[1] Wang Y Han KJ Pang XW Vaughan HA Qu W Dong XY. Large Scale Identification of Human Hepatocellular Carcinoma-Associated Antigens by Autoantibodies. J Immunol (2002) 169:1102–9. doi: 10.4049/jimmunol.169.2.1102 12097419

[2] Milton A Luoto K Ingram L Munro S Logan N Graham AL. A Functionally Distinct Member of the DP Family of E2F Subunits. Oncogene (2006) 25:3212–8. doi: 10.1038/sj.onc.1209343 16418725

[3] Qiao H Di Stefano L Tian C Li YY Yin YH Qian XP. Human TFDP3, a Novel DP Protein, Inhibits DNA Binding and Transactivation by E2F. J Biol Chem (2007) 282:454–66. doi: 10.1074/jbc.M606169200 17062573

[4] Helin K Wu CL Fattaey AR Lees JA Dynlacht BD Ngwu C. Heterodimerization of the Transcription Factors E2F-1 and DP-1 Leads to Cooperative Trans-Activation. Genes Dev (1993) 7:1850–61. doi: 10.1101/gad.7.10.1850 8405995

[5] Huber HE Edwards G Goodhart PJ Patrick DR Huang PS Ivey-Hoyle M. Transcription Factor E2F Binds DNA as a Heterodimer. Proc Natl Acad Sci USA (1993) 90:3525–9. doi: 10.1073/pnas.90.8.3525 PMC46333 8475102

[6] Girling R Partridge JF Bandara LR Burden N Totty NF Hsuan JJ. A New Component of the Transcription Factor DRTF1/E2F. Nature (1993) 362:83–7. doi: 10.1038/362083a0 8446173

[7] Ren LF Ma YY Yue QH Su MQ Hao XK. Effects of TFDP3 on Regulating the Autophagy and Apoptosis of LNCaP Cells. Xi Bao Yu Fen Zi Mian Yi Xue Za Zhi (2012) 28:347–9. 22482402

[8] Pang PH Chan KT Tse LY Chan RC Cheung YK Sin FW. Induction of Cytotoxic T Cell Response Against HCA661 Positive Cancer Cells Through Activation With Novel HLA-A *0201 Restricted Epitopes. Cancer Lett (2007) 256:178–85. doi: 10.1016/j.canlet.2007.06.002 17624664

[9] Hurtado Lopez AM Chen-Liang TH Zurdo M Carrillo-Tornel S Panadero J Salido EJ. Cancer Testis Antigens in Myelodysplastic Syndromes Revisited: A Targeted RNA-Seq Approach. Oncoimmunology (2020) 9:1824642. doi: 10.1080/2162402X.2020.1824642 33101773 PMC7553508

[10] Yin K Liu Y Chu M Wang Y. TFDP3 Regulates Epithelial-Mesenchymal Transition in Breast Cancer. PLoS One (2017) 12:e0170573. doi: 10.1371/journal.pone.0170573 28114432 PMC5256886

[11] DeGregori J Johnson DG. Distinct and Overlapping Roles for E2F Family Members in Transcription, Proliferation and Apoptosis. Curr Mol Med (2006) 6:739–48. doi: 10.2174/1566524010606070739 17100600

[12] Ormondroyd E de la Luna S La Thangue NB. A New Member of the DP Family, DP-3, With Distinct Protein Products Suggests a Regulatory Role for Alternative Splicing in the Cell Cycle Transcription Factor DRTF1/E2F. Oncogene (1995) 11:1437–46. 7478568

[13] Iaquinta PJ Lees JA. Life and Death Decisions by the E2F Transcription Factors. Curr Opin Cell Biol (2007) 19:649–57. doi: 10.1016/j.ceb.2007.10.006 PMC2268988 18032011

[14] Lammens T Li J Leone G De Veylder L. Atypical E2Fs: New Players in the E2F Transcription Factor Family. Trends Cell Biol (2009) 19:111–8. doi: 10.1016/j.tcb.2009.01.002 PMC2808192 19201609

[15] Jiao Y Ding L Chu M Wang T Kang J Zhao X. Effects of Cancer-Testis Antigen, TFDP3, on Cell Cycle Regulation and its Mechanism in L-02 and HepG2 Cell Lines *In Vitro* . PLoS One (2017) 12:e0182781. doi: 10.1371/journal.pone.0182781 28797103 PMC5552311

[16] Ma Y Xin Y Li R Wang Z Yue Q Xiao F. TFDP3 was Expressed in Coordination With E2F1 to Inhibit E2F1-Mediated Apoptosis in Prostate Cancer. Gene (2014) 537:253–9. doi: 10.1016/j.gene.2013.12.051 24406621

[17] Ingram L Munro S Coutts AS La Thangue NB. E2F-1 Regulation by an Unusual DNA Damage-Responsive DP Partner Subunit. Cell Death Differ (2011) 18:122–32. doi: 10.1038/cdd.2010.70 PMC3131880 20559320

[18] Jones PA Ohtani H Chakravarthy A De Carvalho DD. Epigenetic Therapy in Immune-Oncology. Nat Rev Cancer (2019) 19:151–61. doi: 10.1038/s41568-019-0109-9 30723290

[19] Stevens C La Thangue NB. E2F and Cell Cycle Control: A Double-Edged Sword. Arch Biochem Biophys (2003) 412:157–69. doi: 10.1016/S0003-9861(03)00054-7 12667479

[20] Zhang Y Chellappan SP. Cloning and Characterization of Human DP2, a Novel Dimerization Partner of E2F. Oncogene (1995) 10:2085–93. 7784053

[21] Allen KE de la Luna S Kerkhoven RM Bernards R La Thangue NB. Distinct Mechanisms of Nuclear Accumulation Regulate the Functional Consequence of E2F Transcription Factors. J Cell Sci 110 (Pt (1997) 22):2819–31. doi: 10.1242/jcs.110.22.2819 9427290

[22] Hiebert SW Packham G Strom DK Haffner R Oren M Zambetti G. E2F-1:DP-1 Induces P53 and Overrides Survival Factors to Trigger Apoptosis. Mol Cell Biol (1995) 15:6864–74. doi: 10.1128/MCB.15.12.6864 PMC230941 8524253

[23] Shan B Lee WH. Deregulated Expression of E2F-1 Induces S-Phase Entry and Leads to Apoptosis. Mol Cell Biol (1994) 14:8166–73. doi: 10.1128/MCB.14.12.8166 PMC359355 7969153

[24] Hitchens MR Robbins PD. The Role of the Transcription Factor DP in Apoptosis. Apoptosis (2003) 8:461–8. doi: 10.1023/A:1025586207239 12975577

[25] Chu M Yin K Dong Y Wang P Xue Y Zhou P. TFDP3 Confers Chemoresistance in Minimal Residual Disease Within Childhood T-Cell Acute Lymphoblastic Leukemia. Oncotarget (2017) 8:1405–15. doi: 10.18632/oncotarget.13630 PMC5352064 27902457

[26] Bates S Phillips AC Clark PA Stott F Peters G Ludwig RL. P14arf Links the Tumour Suppressors RB and P53. Nature (1998) 395:124–5. doi: 10.1038/25867 9744267

[27] Tian C Lv D Qiao H Zhang J Yin YH Qian XP. TFDP3 Inhibits E2F1-Induced, P53-Mediated Apoptosis. Biochem Biophys Res Commun (2007) 361:20–5. doi: 10.1016/j.bbrc.2007.06.128 17632080

[28] Ding LY Chu M Jiao YS Hao Q Xiao P Li HH. TFDP3 Regulates the Apoptosis and Autophagy in Breast Cancer Cell Line MDA-MB-231. PLoS One (2018) 13:e0203833. doi: 10.1371/journal.pone.0203833 30235236 PMC6147432

[29] Dalton S. Cell Cycle Regulation of the Human Cdc2 Gene. EMBO J (1992) 11:1797–804. doi: 10.1002/j.1460-2075.1992.tb05231.x PMC556637 1582409

[30] Hiebert SW Blake M Azizkhan J Nevins JR. Role of E2F Transcription Factor in E1A-Mediated Trans Activation of Cellular Genes. J Virol (1991) 65:3547–52. doi: 10.1128/jvi.65.7.3547-3552.1991 PMC241350 1828272

[31] Shirodkar S Ewen M DeCaprio JA Morgan J Livingston DM Chittenden T. The Transcription Factor E2F Interacts With the Retinoblastoma Product and a P107-Cyclin A Complex in a Cell Cycle-Regulated Manner. Cell (1992) 68:157–66. doi: 10.1016/0092-8674(92)90214-W 1531040

[32] La Thangue NB. DP and E2F Proteins: Components of a Heterodimeric Transcription Factor Implicated in Cell Cycle Control. Curr Opin Cell Biol (1994) 6:443–50. doi: 10.1016/0955-0674(94)90038-8 7917337

[33] Kent LN Leone G. The Broken Cycle: E2F Dysfunction in Cancer. Nat Rev Cancer (2019) 19:326–38. doi: 10.1038/s41568-019-0143-7 31053804

[34] Lindeman GJ Gaubatz S Livingston DM Ginsberg D. The Subcellular Localization of E2F-4 Is Cell-Cycle Dependent. Proc Natl Acad Sci USA (1997) 94:5095–100. doi: 10.1073/pnas.94.10.5095 PMC24637 9144196

[35] Magae J Wu CL Illenye S Harlow E Heintz NH. Nuclear Localization of DP and E2F Transcription Factors by Heterodimeric Partners and Retinoblastoma Protein Family Members. J Cell Sci 109 (Pt (1996) 7):1717–26. doi: 10.1242/jcs.109.7.1717 8832394

[36] Bertoli C Skotheim JM de Bruin RA. Control of Cell Cycle Transcription During G1 and S Phases. Nat Rev Mol Cell Biol (2013) 14:518–28. doi: 10.1038/nrm3629 PMC4569015 23877564

[37] Henley SA Dick FA. The Retinoblastoma Family of Proteins and Their Regulatory Functions in the Mammalian Cell Division Cycle. Cell Div (2012) 7:10. doi: 10.1186/1747-1028-7-10 22417103 PMC3325851

[38] Kwon JS Everetts NJ Wang X Wang W Della Croce K Xing J. Controlling Depth of Cellular Quiescence by an Rb-E2F Network Switch. Cell Rep (2017) 20:3223–35. doi: 10.1016/j.celrep.2017.09.007 PMC6571029 28954237

[39] Yao G Lee TJ Mori S Nevins JR You L. A Bistable Rb-E2F Switch Underlies the Restriction Point. Nat Cell Biol (2008) 10:476–82. doi: 10.1038/ncb1711 18364697

[40] Ginsberg D. E2F1 Pathways to Apoptosis. FEBS Lett (2002) 529:122–5. doi: 10.1016/S0014-5793(02)03270-2 12354623

[41] DeGregori J Leone G Miron A Jakoi L Nevins JR. Distinct Roles for E2F Proteins in Cell Growth Control and Apoptosis. Proc Natl Acad Sci USA (1997) 94:7245–50. doi: 10.1073/pnas.94.14.7245 PMC23805 9207076

[42] Kowalik TF DeGregori J Leone G Jakoi L Nevins JR. E2F1-Specific Induction of Apoptosis and P53 Accumulation, Which Is Blocked by Mdm2. Cell Growth Differ (1998) 9:113–8. 9486847

[43] Shats I Deng M Davidovich A Zhang C Kwon JS Manandhar D. Expression Level Is a Key Determinant of E2F1-Mediated Cell Fate. Cell Death Differ (2017) 24:626–37. doi: 10.1038/cdd.2017.12 PMC5384025 28211871

[44] Hershko T Chaussepied M Oren M Ginsberg D. Novel Link Between E2F and P53: Proapoptotic Cofactors of P53 Are Transcriptionally Upregulated by E2F. Cell Death Differ (2005) 12:377–83. doi: 10.1038/sj.cdd.4401575 15706352

[45] Powers JT Hong S Mayhew CN Rogers PM Knudsen ES Johnson DG. E2F1 Uses the ATM Signaling Pathway to Induce P53 and Chk2 Phosphorylation and Apoptosis. Mol Cancer Res (2004) 2:203–14. 15140942

[46] Furukawa Y Nishimura N Furukawa Y Satoh M Endo H Iwase S. Apaf-1 Is a Mediator of E2F-1-Induced Apoptosis. J Biol Chem (2002) 277:39760–8. doi: 10.1074/jbc.M200805200 12149244

[47] Irwin M Marin MC Phillips AC Seelan RS Smith DI Liu W. Role for the P53 Homologue P73 in E2F-1-Induced Apoptosis. Nature (2000) 407:645–8. doi: 10.1038/35036614 11034215

[48] Phillips AC Ernst MK Bates S Rice NR Vousden KH. E2F-1 Potentiates Cell Death by Blocking Antiapoptotic Signaling Pathways. Mol Cell (1999) 4:771–81. doi: 10.1016/S1097-2765(00)80387-1 10619024

[49] Sun HX Xu Y Yang XR Wang WM Bai H Shi RY. Hypoxia Inducible Factor 2 Alpha Inhibits Hepatocellular Carcinoma Growth Through the Transcription Factor Dimerization Partner 3/E2F Transcription Factor 1-Dependent Apoptotic Pathway. Hepatology (2013) 57:1088–97. doi: 10.1002/hep.26188 PMC3594482 23212661

[50] Chan RC Pang XW Wang YD Chen WF Xie Y. Transduction of Dendritic Cells With Recombinant Adenovirus Encoding HCA661 Activates Autologous Cytotoxic T Lymphocytes to Target Hepatoma Cells. Br J Cancer (2004) 90:1636–43. doi: 10.1038/sj.bjc.6601706 PMC2409703 15083197

[51] Cafri G Gartner JJ Zaks T Hopson K Levin N Paria BC. mRNA Vaccine-Induced Neoantigen-Specific T Cell Immunity in Patients With Gastrointestinal Cancer. J Clin Invest (2020) 130:5976–88. doi: 10.1172/JCI134915 PMC7598064 33016924

[52] Tovar H Garcia-Herrera R Espinal-Enriquez J Hernandez-Lemus E. Transcriptional Master Regulator Analysis in Breast Cancer Genetic Networks. Comput Biol Chem (2015) 59 Pt B:67–77. doi: 10.1016/j.compbiolchem.2015.08.007 26362298

[53] Zhang D Zhou S Liu B. Identification and Validation of an Individualized EMT-Related Prognostic Risk Score Formula in Gastric Adenocarcinoma Patients. BioMed Res Int (2020) 2020:7082408. doi: 10.1155/2020/7082408 32309437 PMC7142392

